# The Gut Microbiome of 54 Mammalian Species

**DOI:** 10.3389/fmicb.2022.886252

**Published:** 2022-06-16

**Authors:** Nadieh de Jonge, Benjamin Carlsen, Mikkel Hostrup Christensen, Cino Pertoldi, Jeppe Lund Nielsen

**Affiliations:** ^1^Department of Chemistry and Bioscience, Aalborg University, Aalborg, Denmark; ^2^Aalborg Zoo, Aalborg, Denmark

**Keywords:** gut microbiota, diet, captivity, gut physiology, mammals, conservation biology

## Abstract

The gut microbiome plays a critical role in many aspects of host life, and the microbial community composition is heavily influenced by the prevailing conditions in the gut environment. Community composition has been suggested to have large implications for conservation efforts, and gut health has become of interest for optimizing animal care in captivity. In this study, we explore the gut microbiome of a wide range of animals in the context of conservation biology. The composition of the gut microbial community of 54 mammalian animal species was investigated using 16S rRNA gene amplicon sequencing. The composition of the gut microbiota clearly reflects diet and the structure of the gastrointestinal system, and it is to a certain degree more similar between closely related animals. Specific clusters of taxa were observed across animals of the same species, diet, and gut morphology. The microbiota retained regardless of captivity status is hypothesized to cover important symbiotic relationships with the host, while the remaining part reflects the artificial living conditions and can therefore be used as a future tool for conservation biologists. For five animal species (giraffes, horses, baboons, elephants, and zebras), it was possible to compare the microbiota of wild and captive individuals. Differences were observed in the proportion of microbiota detected between wild and captive specimens of the same animal species. We propose that the gut microbiota harbours important species, which can potentially serve as indicators for the well-being of the animal and the effect of living in captivity.

## Introduction

The gut microbiome has an important role in relation to the health and well-being of the host, primarily for its role in regulating nutrient and energy uptake through assisting in the energy uptake by facilitating digestion of complex food items (Stevens and Hume, 1998). But the microbiome has also been linked to other processes such as the development and maintenance of the immune system, behaviour, and reproduction (Kamada et al., 2013; McFall-Ngai et al., 2013; Bahrndorff et al., 2016; Pickard et al., 2017). Furthermore, the gut microbiome also protects the host from the establishment of potential pathogens (Pickard et al., 2017; Kogut et al., 2020), thereby functioning as a barrier. This symbiotic relationship has co-evolved over numerous generations, and the composition of the microbiome is influenced by factors relating to host physiology and ecology, as well as other biotic and abiotic environmental factors (McFall-Ngai et al., 2013; Moeller and Sanders, 2020).

Conservation efforts such as breeding or rehabilitation have a significant impact on the lives of the animals concerned (Bahrndorff et al., 2016). Animals living in captivity often experience extreme conditions, including an altered diet and social structure, compared to the environment in which they co-evolved (Redford et al., 2012). Furthermore, in human-made environments, increased population densities, contact with humans, medical treatment, and antibiotic administration in particular are also introduced (Kirkwood, 2003). All of these changes can contribute to the phenomena of selective pressure (pressure relaxation or increased pressure from altered diet composition) and can influence the composition of the gut microbiota and potentially cause negative effects on the health of animal health (Redford et al., 2012; Hauffe and Barelli, 2019). In addition, inbreeding has also been shown to have a negative impact on many aspects of animal life, including the gut microbiota (Bahrndorff et al., 2016). It is hypothesized that a better understanding of the effects of captivity on an animal's microbiome is important for providing optimal care, specifically in regards to health and general welfare (Bahrndorff et al., 2016; Hauffe and Barelli, 2019).

The gastrointestinal system and co-habiting microbiota of vertebrate hosts have co-evolved alongside the host, becoming specialized to the nutritional needs and preferences of each species (Stevens and Hume, 1998; Moeller and Sanders, 2020). The earliest evolved mammals had a simple gut system, a gut morphology that is still observed in many animals with a meat-based dietary preference such as representatives of the order Carnivora (Van Valkenburgh, 2007), as well as a number of omnivores including pigs and humans (Stevens and Hume, 1998). Animals are not able to produce the endogenous cellulolytic enzymes required to digest plant-based materials and rely on symbiosis with their gut microbiota to achieve this, as well as similar enzyme-dependent tasks, leading to a more complex gastrointestinal tract development (and microbiota) compared to carnivores (Ley et al., 2008; Bayané and Guiot, 2011). Strict herbivores can digest their food by either hindgut (e.g., horses and elephants) or foregut fermentation (e.g., ruminants such as giraffes and kangaroos) (Stevens and Hume, 1998; Bayané and Guiot, 2011). Gut morphology and dietary preferences cannot be strictly divided into specific orders of animals, as some species within the same order have developed specialized dietary strategies (and thus microbiota) over time (Stevens and Hume, 1998; McFall-Ngai et al., 2013). Prominent examples of this type of specialization are the giant pandas, which consume a strictly herbivoric diet while possessing a simple gut morphology (Van Valkenburgh, 2007; Li et al., 2015), and the koala, which lives almost exclusively on foliage from the genus *Eucalyptus* (Brice et al., 2019).

High-throughput sequencing technology has made it possible to gain insight into the gut microbiota of a diverse range of animal species, including primates (Amato et al., 2013; Clayton et al., 2016; Greene et al., 2021), carnivores (An et al., 2017), reptiles (Tang et al., 2020), and birds (Roggenbuck et al., 2014; García-Amado et al., 2018), among others. The majority of all microbiome studies have been conducted on single species, which provide excellent conditions for investigating short-term exposure effects such as diet changes. Studies across multiple species, on the other hand, allow investigations into evolutionary dependencies such as the effects of phylogenetic traits (including gut morphology) (Ley et al., 2008) and universal feeding strategies. Important parameters for shaping the gut microbiota composition in individual animal species have been shown to include dietary preferences (Muegge et al., 2011; Poulsen et al., 2017), and to a lesser degree, also factors such as sex, social interaction, biogeography, and the individual's genetic profile (Yatsunenko et al., 2012; Moeller et al., 2013; Song et al., 2013; Yuan et al., 2015; Eisenhofer et al., 2021).

A number of studies have compared the microbiota of wild and captive animals. Studies in diverse animal species, including Antarctic seals (Nelson et al., 2013), various primates (Amato et al., 2013; Hale et al., 2019; Greene et al., 2021), and horses (*Equus ferus caballus*) (Metcalf et al., 2017) showed significant differences in the composition of the gut microbiota in wild individuals compared to captive relatives. Another study showed that primates living in captivity gain a more human-like microbiota composition over time (Clayton et al., 2016), and that this is reproducible between captive populations (Houtz et al., 2021). These results support the hypothesis that transfer of animals from their natural habitat to captivity can induce rapid and extensive changes to the gut microbiota composition and thereby affect other aspects of animal welfare. However, many unanswered questions relating to the effect of habitat changes on gut microbiota in animals still exist, as a meta-analysis study has also shown that the differences between wild and captive microbiomes are heterogeneously distributed and cannot simply be generalized across species (Alberdi et al., 2021).

In studies investigating a large number of animal species, it is possible to compare microbiota between groups or habitats (Ley et al., 2008; McKenzie et al., 2017; O'Donnell et al., 2017; Youngblut et al., 2019; Alberdi et al., 2021). A frequent observation has been a reduced microbial diversity in captive individuals compared to their wild counterparts in some animal species (Kohl et al., 2014), while others have observed species-specific differences (McKenzie et al., 2017; Alberdi et al., 2021). Besides changes to the overall microbiota composition in the gut of captive animals, the functional composition of the microbiota has been suggested to be even more important (Lozupone et al., 2012), with species-specific responses potentially determining which animals adapt better to changes than others. However, this aspect of the animal gut microbiome remains largely unexplored (Hauffe and Barelli, 2019). In summary, overarching trends in the microbiota of wild animals and captive relatives have been observed, but many questions related to the specific response of animal gut microbiota to living in captivity and the effects on the host remain unanswered.

The aim of this study was to investigate the gut microbiota of mammalian animal species and across a wide cohort to explore the influence of phylogeny, gut morphology, and dietary choices on the microbial community composition. The microbiomes of 54 mammalian species from captive and wild habitats were analyzed using 16S rRNA gene amplicon sequencing, and alpha and beta diversity indices between the different animal species were explored in detail. Differences in the gut microbiomes of animals of the same species living in captivity were compared, as well as that of captive animals and their wild relatives to investigate the effects of captivity.

## Methods

### Sample Collection

Samples from animals living in captivity at four different zoos in Denmark (*n* = 52) and Norway (*n* = 4) were collected in the period of October 2016 to July 2018. Fecal deposits were collected within 1 h of deposition, and where possible, only the interior of the fecal deposit was sampled to limit potential contamination. Samples were transported directly to the laboratory on ice, or when shipped cross-border, stored in RNAlater (Sigma-Aldrich), and immediately stored at −18°C upon arrival. In addition, samples from wild animals were obtained from various locations in Denmark, in Tanzania, and in Zimbabwe (*n* = 22). These samples were collected based on normal appearance (color, odor, and consistency) and the criteria that no significant evidence of exposure to the environment and dehydration were visible, and they were transported in 96% ethanol and under cold conditions where possible. An overview of all samples included in the study is shown in Supplementary Table S1. All samples were stored at −18°C until further processing.

Ethical approval for obtaining animal fecal samples was not required as per national guidelines. A written statement from Aalborg Zoo's Veterinary Service was obtained, declaring that no animals experienced any break from disturbances in their daily routines during sampling for this study.

### DNA Extraction

All samples were gently homogenized by stirring to obtain a representative subsample of approximately 0.5 g. Total genomic DNA was extracted using the FastDNA Spin kit for soil (MP Biomedicals) following the manufacturer's instructions, with hot phenol pre-treatment as described elsewhere (Albertsen et al., 2013). The quality of the DNA extracts was assessed using a TapeStation 2200 and Genomic DNA ScreenTapes (Agilent), and their concentration was estimated using a Qubit 3.0 fluorometer and Qubit dsDNA HS Assay Kit (Thermo Fisher Scientific).

### 16S rRNA Gene Amplicon Sequencing

The amplicon sequencing approach using the 16S ribosomal RNA gene as a phylogenetic marker was chosen as it provides an efficient and cost-effective approach for microbiome analysis. The hypervariable V4 region of the 16S rRNA gene was amplified using the welldescribed primer sets 515F GTGCCAGCMGCCGCGGTAA and 806R GGACTACHVGGGTWTCTAAT (Caporaso et al., 2011) fused with Illumina adapters. Genomic DNA (10 ng) was amplified in 25 μL duplicate PCR reactions, as previously described (Bahrndorff et al., 2018). Equimolar amounts of all sample libraries were sequenced on an Illumina MiSeq platform using reagent kit v3 (2 × 300 PE) and a 20 % Phi-X spike-in. Raw sequence data were treated using the AmpProc pipeline (v5.1) (https://github.com/eyashiro/AmpProc). In brief, USEARCH11 was used for read quality filtering, PhiX removal, chimeric and spurious read removal, and merging of paired-end reads (Edgar, 2010). Amplicon sequencing variants (ASVs) were generated using the UNOISE3 algorithm (Edgar, 2016b). Taxonomy was assigned using SINTAX (Edgar, 2016a) and using SILVA release S138 as the reference database (Quast et al., 2013).

### Data Analysis

The microbial community data were analyzed using R version 4.0.2 (R Development Core Team, 2021), with RStudio version 1.3.959 (www.rstudio.com). Alpha and beta diversities were explored using the package ampvis2 (Andersen et al., 2018), and the clustered heatmap was generated using the packages vegan (Oksanen et al., 2016) and gplots (Warnes et al., 2019). All other visualizations were created using the package ggplot2 (Wickham, 2016). Alpha diversity was measured using the ChaoI index (Chao, 1984), and beta diversity was estimated using non-metric multi-dimensional scaling (NMDS) on Bray-Curtis distances (Bray and Curtis, 1957). Differences in alpha diversity between groups were tested using the Wilcoxon ranked sum test with Benjamini–Hochberg correction for multiple testing, and differences in beta diversity between groups were tested using PERMANOVA and ANOSIM with 999 permutations. A clustered heatmap was generated using Ward agglomerative clustering (Murtagh and Legendre, 2014) on Bray-Curtis distances of raw abundance data. All quantitative data for sequences and ASVs are presented as mean ± standard deviations.

## Results

### Sequence Quality

Sequencing of 76 animal fecal samples generated a grand total of 3,304,384 reads. Sequencing depth was examined using rarefaction curves (Supplementary Figure S1), and a minimum number of 7,500 sequences per sample were determined to be sufficient for further downstream analysis. After quality filtering, 70 samples entered the microbial community analysis, of which 54 were from captive animals and 16 from wild animals, with an average of 47,096 ± 36,399 reads per sample.

### Alpha Diversity of Captive Animal Species

**Figure 1:**
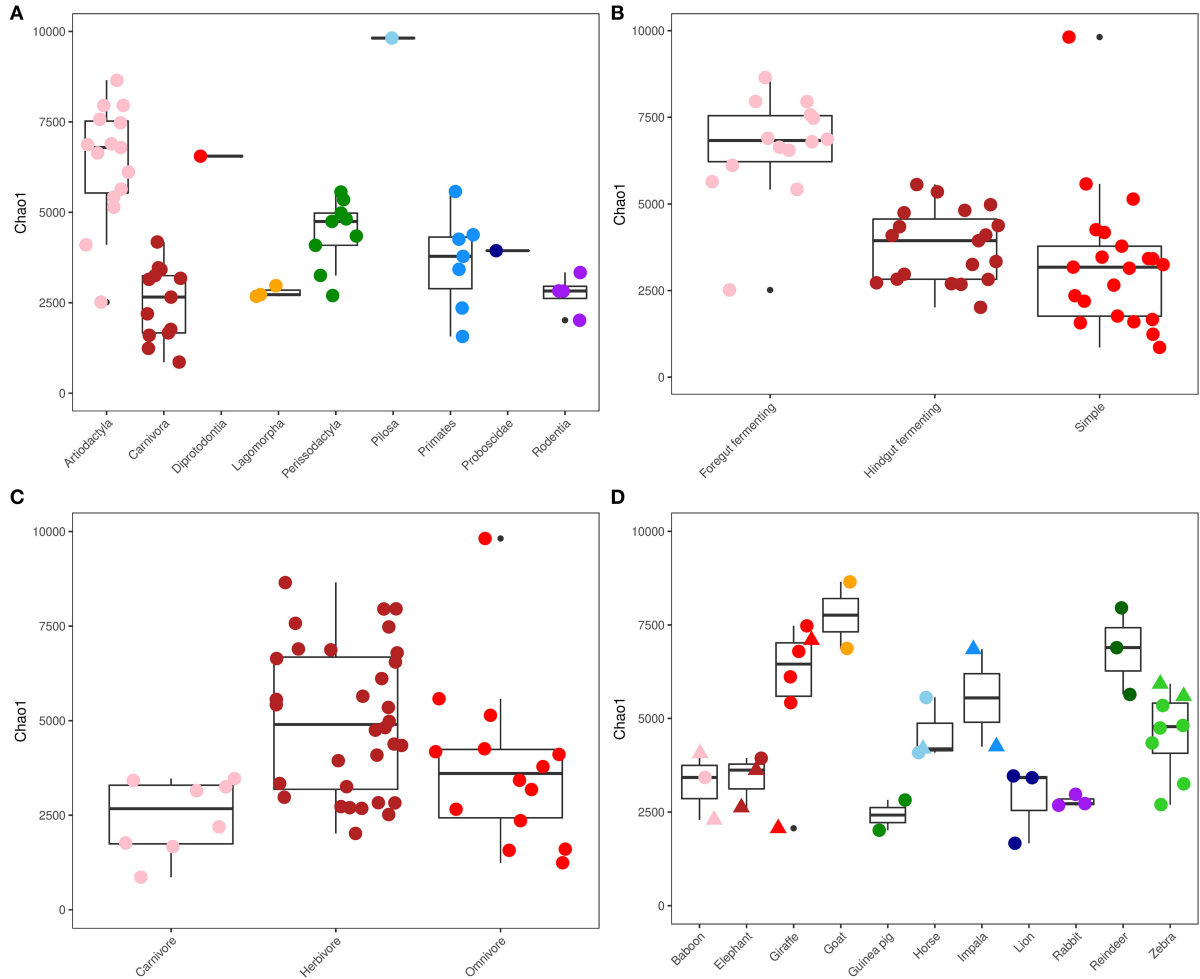
Alpha diversity. ChaoI diversity index measurements for captive mammals, sorted and colored by phylogeny **(A)**, gut morphology **(B)**, diet **(C)**, and microbiomes of animals with multiple specimens living in captivity (circle) and in the wild (triangle) **(D)**. Data are displayed as boxplots that bound the interquartile range (IQR) divided by the median and whiskers that extend 1.5 × IQR past the box. Outliers are shown as solid black points.

The richness of the gut microbial communities of the captive animals was measured using the observed number of ASVs and the Chao1 index (Figure 1). The average ratio between the observed and estimated number of ASVs (Chao1) in each sample was 0.76 ± 0.07, which indicates that the majority of the diversity in the gut microbiota had been captured (Supplementary Table S2). The highest richness was seen in the sample from the order Pilosa (Giant anteater), with 6,440 ASVs (Figure 1A). Overall, the orders Perissodactyla (*n* = 9) and Artiodactyla (*n* = 15) contained a high gut microbial richness, compared to the order Carnivora (*n* = 13) which had a significantly lower richness per sampled animal (*p* <0.05). Foregut fermenting animals (*n* = 14) had the overall highest gut microbiota richness within the different gut morphologies (Figure 1B), while in the animals with a simple gut physiology (*n* =21), the lowest overall microbial diversity was observed. Foregut fermenting animals had a significantly higher microbiota diversity, compared to the hindgut fermenters and simple gut groups (*p* <0.0001). Sorted by dietary choices (Figure 1C), the herbivores had the highest richness (*n* = 32), while the carnivores had the lowest microbial community diversity (*n* = 8). Estimated microbiota richness was significantly different between the herbivores and carnivores (*p* = 0.004).

### Specific Clusters of Microorganisms Associate With Animals Based on Phylogeny and Diet

**Figure 2:**
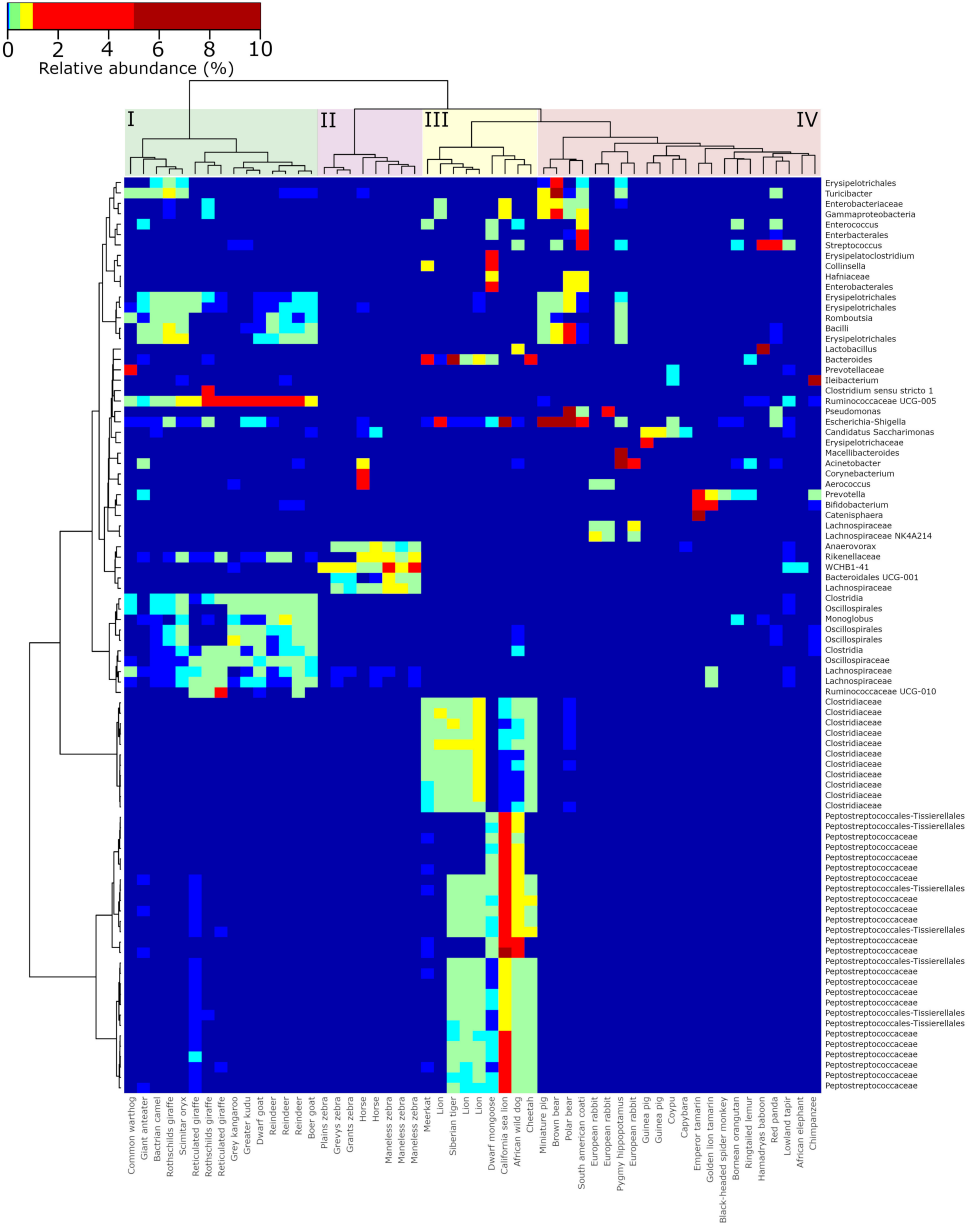
Hierarchically clustered heatmap of the most abundant genera observed in the gut microbiota of captive animals. The 50 most abundant ASVs were selected from samples stemming from herbivores, carnivores, and omnivores, respectively. These ASVs were re-aggregated to the genus level, a distance matrix of Bray-Curtis distance was generated, and clustering was performed using Ward agglomerative clustering. Colored boxes highlight the four major clusters of ruminating herbivores (I), non-ruminating herbivores (II), carnivores (III), and omnivores (IV).

The presence of distinct groups of gut microbes associated with specific groups of animals based on either phylogeny, gut morphology, or diet preference was investigated by generating a hierarchical clustered heatmap of the most abundantly observed microorganisms (the 50 most abundantly observed ASVs in herbivores, omnivores and carnivores; Figure 2). Overall, groupings of ruminants (cluster I), horses (and zebras) (cluster II), carnivores (cluster III), and a mixed cluster of herbivores and omnivores (cluster IV) were formed. An abundant group of microbes was observed to be ubiquitously present in a tightly clustered group of all foregut fermenting animals (cluster I, *n* = 15), including reindeer, giraffes, and goats (Artiodactyla). These included representatives of *Oscillospiraceae, Lachnospiraceae, Monoglobus*, and *Ruminococcaceae* UCG-005. Representatives of the families Clostridiaceae and Peptostreptococcaceae were primarily observed in a group of carnivores (including lions, cheetah, and African wild dog) that were clustered together (cluster III). Omnivoric bears, including the South American coati and red panda, were found on a separate branch from the other members of the mixed cluster of herbivores and omnivores (cluster IV), but directly adjacent to the order Carnivora, and were associated with a cluster of microorganisms including *Turicibacter* and a representative of the *Enterobacteriaceae*. The largest cluster consisted of omnivores such as bears and primates, as well as herbivores such as capybara and guinea pigs (cluster IV). In these samples, *Bifidobacterium, Pseudomonas, Prevotella*, and *Escherichia-Shigella* were observed abundantly (>0.1% of total reads).

### Species Relationships and Traits Shape the Microbiota Composition of Captive Animals

**Figure 3:**
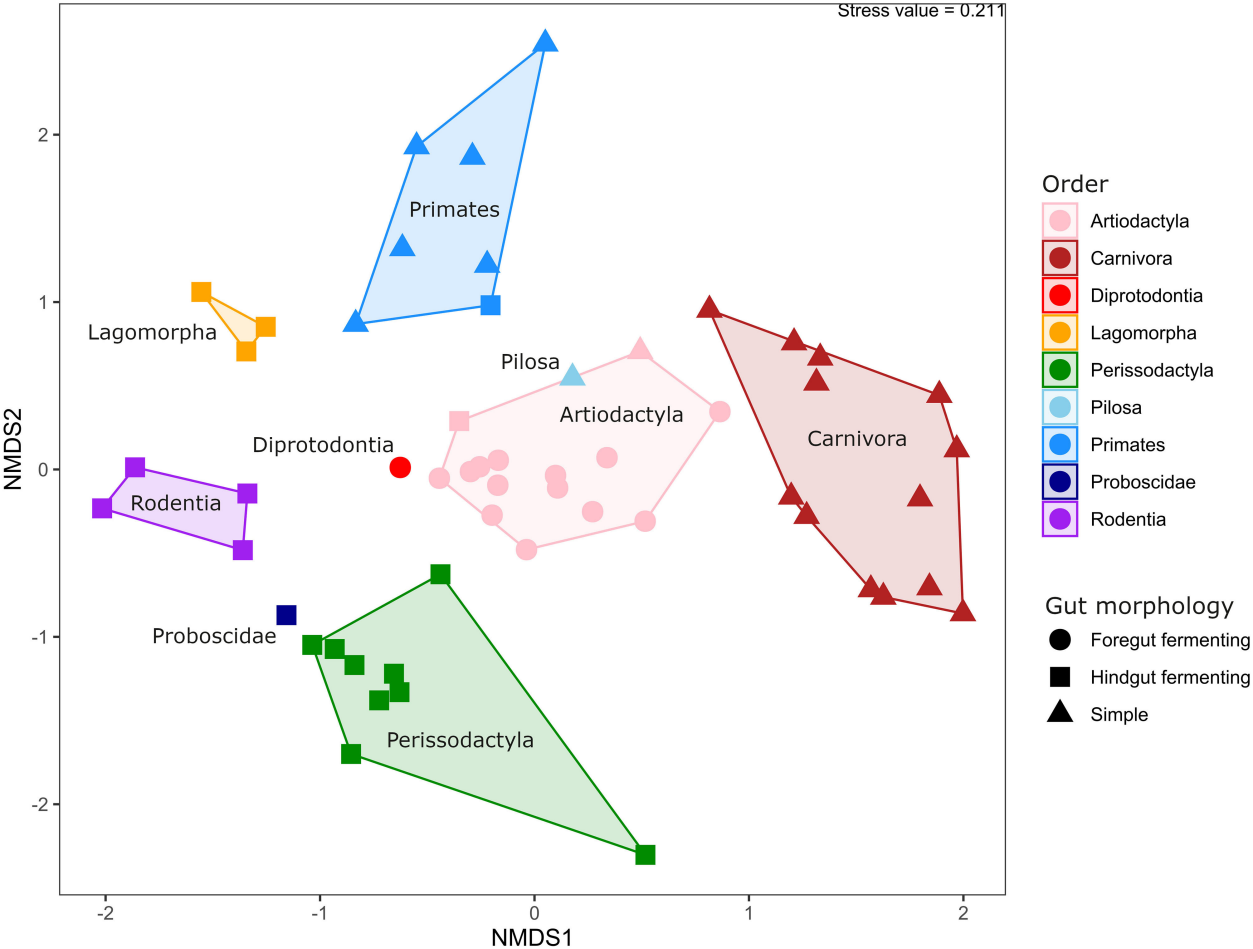
Beta diversity of animals in captivity. Non-metric dimensional scaling analysis based on Bray-Curtis distances, with samples colored by phylogeny and shaped by gut morphology. A polygon is drawn around samples from the same phylogenetic group, and the names of the individual groups are displayed within their respective polygons.

The similarities and differences in gut microbiota composition in 54 animal specimens across 42 species were explored using non-metric, multi-dimensional scaling analysis based on Bray-Curtis distances (Figure 3). Statistical analysis using PERMANOVA showed that the phylogenetic relatedness of the host was the major factor to explain the observed differences (*p* < 0.001, *R*^2^ = 0.83), followed by gut morphology (*p* < 0.001, *R*^2^ = 0.66) and dietary preferences (*p* < 0.001, *R*^2^ = 0.35). This was also visible in the beta diversity analysis, in which all animal orders represented in the study were clearly separated from each other (ANOSIM; *p* < 0.001, *R*^2^ = 0.81). The primarily herbivoric orders Perissodactyla, Proboscidae, Diprodontia, Artiodactyla, and Pilosa were relatively clustered together, while Carnivora, Rodentia, Lagomorpha, and Primates were grouped separately. Animals from the order Artiodactyla and Perissodactyla were clustered closer together within their own groups compared to other animal groups, while the greatest variation in gut microbiota composition was seen in the groups with primates and carnivores.

### Gut Microbiota Differences Between Wild and Captive Animals of the Same Species

This study included 12 animal species (disregarding subspecies) where multiple individuals were sampled, either from different captive habitats or where both wild and captive specimens were included in the study. The difference in measured richness in the gut microbiome of these animals varied; the lowest variation in diversity was observed in rabbit and guinea pigs (Figure 1D), while a greater variation was observed between the sampled zebras and giraffes, both of which included both wild and captive specimens. Beta diversity analysis of the replicated animals (Supplementary Figure S2) showed that the differences between specimens of the same species were small for horses, goats, rabbits, and reindeer, while a greater variation in gut microbiota composition was observed in elephants, lions, zebras, giraffes, and baboons (ANOSIM; *p* < 0.001, *R*^2^ = 0.87). Overall, the sampled captive animals clustered near their respective wild relatives in an NMDS analysis (Supplementary Figure S3) and within their own cluster based on evolutionary relationships, except for one giraffe and one elephant, which were observed near the bottom of the plot, grouping away from the other samples. In addition, the inclusion of the wild animals into the NMDS analysis increased the variation within the individual phylogenetic groups, and the orders Perissodactyla and Proboscidae now overlapped (ANOSIM; *p* < 0.001, *R*^2^ = 0.77).

Captive animals from the same species, regardless of whether they were from the same or a different habitat, generally shared a great similarity in their overall gut microbial community structure (Supplementary Figure S4). This similarity also extended across certain animal species, especially ruminants and other herbivores. The differences between the gut microbial communities of animals from the same species sampled in both their captive and natural habitats (baboons, elephants, giraffes, horses, and zebras) were compared through analysis of their alpha diversity (Figure 1D), as well as the microbiome composition (Supplementary Figure S5). The smallest variation in microbial community composition between individuals of the 5 animals of interest was seen among the horses (Supplementary Figures S2, S4), while a greater variation between individuals was observed for baboons, elephants, giraffes, and zebras, but no clear separation was visible based on captivity status. The greatest similarity in microbial community composition between wild and captive specimens was observed in the horses, zebras, and giraffes (Perissodactyla and Artiodactyla) (Supplementary Figure S5), while a tendency toward greater variation was seen in the elephants and baboons. In both the horses (*n* = 3) and giraffes (*n* = 6), approximately 40 % of the abundantly identified ASVs (≥0.1% of total reads) were observed in both the wild and captive specimens (Supplementary Figure S5D,E). In elephants (*n* = 3) and baboons (*n* = 3) (Supplementary Figures S5B,C), this overlap was a lot smaller, with around 15 and 25% of abundant ASVs shared, respectively.

## Discussion

The aim of this study was to examine the gut microbiota composition of 54 different animal species stemming from wild and captive habitats. High-throughput sequencing of 70 samples yielded high-quality reads covering the majority of the diversity present in the samples, although the rarefaction curves of the analyzed samples (Supplementary Figure S1) did show that not all of the microbiota was captured, and it is likely that the single individuals analyzed for most species are not fully representative for the animal species. This was taken into account by performing the analysis with conservative considerations in mind. The approach used in this study was to sample different animals and compare the differences in gut microbiota among animal species rather than to focus on multiple individuals from a limited number of species. The large number of animal species sampled allowed for cross-species comparisons to gain valuable information regarding phylogeny and feeding strategies. In addition, a number of animal species included in the study were sampled with multiple replications from different captive and wild habitats to explore differences between individuals.

### Microbial Community Composition of Captive Animals Is Shaped by Phylogeny and Diet

Samples representing the orders Artiodactyla and Perissodactyla (*n* = 24 collectively) contained the greatest microbial community diversity (Figure 1), while samples from carnivoran representatives had the lowest richness. Overall, this is in line with the results of a previous study that explored the microbial community of 41 mammals, which also observed a large difference in diversity between the same phylogenetic groups (McKenzie et al., 2017). A herbivoric diet requires a more diverse set of microorganisms to digest efficiently compared to meat-rich diets (Bayané and Guiot, 2011), which explains the increased diversity of the gut microbiome profiles observed for animals with these dietary preferences. The predominant taxa identified in fecal microbiota across all investigated animals, regardless of their diet, were *Firmicutes* and *Bacteroidetes*, which is in line with previous studies (O'Donnell et al., 2017; Youngblut et al., 2019).

Hierarchical clustering analysis revealed specific and abundant associations of *Ruminococcaceae, Oscillospiraceae*, and a few other microbial taxa that were ubiquitously associated with the ruminants in the dataset. These organisms (and others) were also observed abundantly in ruminants in a previous study focused on domesticated animals in Ireland (O'Donnell et al., 2017), as well as in giraffes (Schmidt et al., 2018). Ruminants have a specific gut morphology and metabolism compared to many other animal groups (Stevens and Hume, 1998; Henderson et al., 2015), and it was therefore expected to see a more uniform microbiota composition and less variation between individuals compared to non-ruminants. Adversely, the gut microbiota profiles of animals with carnivoric dietary preferences were the least diverse among the sampled animals (Figure 1). Previous microbiota studies in carnivores, including leopard cats (*Prionailurus bengalensis*), Eurasian otters (*Lutra lutra*), raccoon dogs (*Nyctereutes procyonoides*) (An et al., 2017), and Asiatic black bears (*Ursus thibetanus*). Song et al. (2017) observed a similar fecal microbiota diversity to those observed in this study. The families *Clostridiaceae* and *Peptostreptococcaceae* were specifically associated with sampled carnivores and have often been observed in the fecal microbiota of animals that include meat in their diet (An et al., 2017; Youngblut et al., 2019).

In this study, the gut microbiota composition was influenced by evolutionary history, gut morphology, and diet, in order of importance. This is in line with several previous studies that also examined a larger range of animal species (Ley et al., 2008; McKenzie et al., 2017), but also with studies in which the diet was reported to be a strong predictor of gut microbiota composition (Muegge et al., 2011; Youngblut et al., 2019). Collectively, these studies show that despite focusing on different aspects of gut microbiota, microbial community analyses in animals have strong predictive capabilities.

### Comparison of Gut Microbiome Community of Captive and Wild Animals

The gut microbiota profiles of animal species where multiple individuals and both wild and captive species were sampled (baboons, elephants, giraffes, horses, and zebras) were examined in detail (Figure 1D, Supplementary Figures S2–S5). Some variation in the microbiome of sampled individuals of the same species was observed, but no significant correlational tendencies were identified based on either phylogeny or dietary preferences of the analyzed species. This is in line with a recent study comparing many different microbiota samples in different vertebrates, which found that differences between wild and captive individuals were heterogeneously distributed among species (Alberdi et al., 2021). Individual variation in the gut microbiome is determined by many factors (Pascoe et al., 2017), and the limited number of replications and samples from individual organisms investigated in this study was not sufficient to reflect on this aspect. However, retention of microbiota did show an interdependency for the animal species where both captive and wild individuals were sampled. Horses and giraffes showed a greater overlap in the microbial consortia observed in individuals from either habitat compared to zebras and elephants. Previous studies in horses and giraffes have likewise shown a high microbial community diversity in these animals (Zhao et al., 2016; Schmidt et al., 2018). However, horses have also been shown to contain lower microbial diversity in their fecal microbiota in captivity compared to wild individuals (Metcalf et al., 2017). One of the most divergently observed microorganisms was *Fibrobacter*, a microbe associated with fiber content in lignocellulosic biomass and prominently observed in animals consuming a plant-based diet (Neumann et al., 2017). The highly diverse and specialized microbiota of animals with herbivoric diets, and ruminating animals in particular, may be more resilient to changes due to their essential function in the degradation of lignocellulosic materials that cannot be achieved without microbial symbiosis (Bayané and Guiot, 2011).

The greatest divergence in gut microbiota composition between wild and captive individuals was observed in elephants. Furthermore, a large proportion of the microbiota found in the baboons was divergent between wild and captive specimens, suggesting altered microbiota. This is in agreement with recent studies where a strong correlation was found between gut microbiota and food availability, habitat and dietary composition in primates and elephants (Nakamura et al., 2011; Clayton et al., 2016; McKenzie et al., 2017; Kartzinel et al., 2019; Zhang et al., 2019). Among the most differentially abundant organisms were the genera *Collinsella* (abundant in the wild specimens) and *Lactobacillus* (abundant in captive individuals). These genera have been associated with dietary fiber content (Barrett et al., 2018) and dairy products (Azad et al., 2018), respectively, suggesting that the dietary composition between these sampled individuals may have played a large role in the observed differences.

### Gut Microbiota Studies in Wild and Captive Animals

An increasing number of studies have investigated the gut microbiota of various animals in natural and captive habitats (McKenzie et al., 2017; Pascoe et al., 2017; Youngblut et al., 2019). One of the globally occurring tendencies across animal microbiome studies has been a trend toward lower gut microbial community richness among captive animals compared to their wild relatives. However, cross-examination of several studies also showed that microbiota diversity changes between wild and captive individuals are not evenly distributed across all species (Alberdi et al., 2021). Decreasing microbial richness has been linked to differences in the diversity and availability of food items in bears (Borbón-García et al., 2017), as well as a selection of African megafauna (Kartzinel et al., 2019). The observed differences could also relate to the increased contact with humans and modern human food items with little variations among captive primates that slowly cause humanization of the gut microbiota (Clayton et al., 2016), something that has also been shown to be both predictable and reproducible in non-human primates (Houtz et al., 2021). Based on these and other studies, it can be hypothesized that the overall changes to the host microbiota are generally more profound when an animal lives in a fully human-made environment, compared to a semi-controlled environment such as a nature reserve. A significantly changed microbiome could also compromise animal health, as the gut microbiota are also known to protect against pathogenic bacteria that are ingested with food items, e.g., dead prey (Roggenbuck et al., 2014; Blumstein et al., 2017). Furthermore, it has recently been shown that certain predators are able to metabolize bacterial toxins through their microbiota (Levin et al., 2021), which further highlights the contribution of host–microbe symbiosis to the functional capabilities of the microbiome in the gut.

The data obtained in this study also indicates that it is possible to identify microorganisms in animal microbiome data that are of potential importance to the host regardless of captivity status. Microbiota retained in both wild and captive individuals may provide valuable information about the symbiotic dependencies of the well-being of an animal (McKenzie et al., 2017). In horses, giraffes, and zebras, up to 50% of the identified ASVs were found in both wild and captive animals, while it was only 20% in elephants (Supplementary Figure S5). A distinct difference could also be seen in some of the abundant microbes found in the baboons (Supplementary Figure S5B), suggesting a potential shift in important microbiota. Previous studies have shown that large primates in captivity evolve a more human-like microbiota over time, primarily due to a change in diet (Clayton et al., 2016). Identification of keystone microbes in the gut microbiota can serve as indicators of the host's adaptation to the surrounding environment. Furthermore, monitoring of keystone organisms of high importance for not only the gut microbiome composition but also its metabolic functional potential can provide important new tools in conservation efforts. However, identification of keystone organisms cannot be based on microbiota comparisons alone; information regarding temporal dynamics, function, activity, and a given microbe's interconnectedness within the microbiome are needed in order to identify true keystone organisms from a given ecosystem (Banerjee et al., 2018).

### The Potential of Microbial Community Data in Conservation Efforts

The findings from this study, as well as the previous animal microbiota studies collectively, support the proposed link between animal health, responses to the environment, and gut microbiota composition (McKenzie et al., 2017; Metcalf et al., 2017; Hauffe and Barelli, 2019; Stothart et al., 2019; Levin et al., 2021), providing evidence that the gut microbiome of animals can provide information about different aspects of host welfare. This could potentially serve as an important tool for future conservation biologists.

Non-invasive fecal microbial community analysis also have the potential to become a valuable toolbox for monitoring the health and general well-being of animals living in captivity (Redford et al., 2012; Stumpf et al., 2016). It has been suggested that the multitude of studies surrounding the gut microbiota in humans and model organisms can also be extended to other animal species, to provide a framework for monitoring of animal microbiota in relation to host health and disease (Amato, 2016; Bahrndorff et al., 2016). This framework would need to be based on individual species knowledge regarding temporal variation and stability of the microbial community in order to elucidate changes in gut microbiota and discern meaningful information regarding the host. Monitoring gut composition could be used to assess environmental stress and determine whether semi- or fully man-made environments such as natural parks and zoos are adequate for the animal. Future studies might also reveal how gut microbiota monitoring can provide important information on habitat fractionation, urbanization and climate change. In addition, it has also been suggested to consider the microbiome as a source of adaptive potential instead (Hauffe and Barelli, 2019). The results of this study support the potential of applying microbiome data in animal welfare and conservation processes, but additional data regarding animal diet, behaviour, health, and other parameters are needed to support microbial community data in order to make meaningful inference in relation to conservation.

## Conclusion

This study showed that gut microbiota composition in mammalian animals is driven by phylogenetic relatedness, gut morphology, and diet, as well as by large environmental differences in terms of living in captivity or in the wild. The presence of animal or diet-specific microbes and the retention of microbiota between captive and wild animals of the same species suggest that keystone microbes are present in the animal gut, and that these may be retained regardless of habitat status. Differences in the proportion of microbiota retention between animal species have implications for the monitoring of (gut) health in animals in captivity. Our findings support the potential and importance of gut microbiota analysis in current conservation efforts as an additional measure of animal welfare and health.

## Data Availability Statement

The datasets presented in this study can be found in online repositories. The names of the repository/repositories and accession number(s) can be found at: https://www.ebi.ac.uk/ena, PRJEB36809.

## Author Contributions

JN conceived the ideas, designed the work, and achieved the funding with contributions from NJ and CP. BC and MC did the sampling and laboratory work with guidance from NJ. NJ analyzed the data with guidance from JN. NJ and JN led the writing of the manuscript with contributions from CP, BC, and MC.

## Funding

The Aalborg Zoo Conservation Foundation (AZCF) supported this research (Grant Number: 3-2017).

## Conflict of Interest

The authors declare that the research was conducted in the absence of any commercial or financial relationships that could be construed as a potential conflict of interest.

## Publisher's Note

All claims expressed in this article are solely those of the authors and do not necessarily represent those of their affiliated organizations, or those of the publisher, the editors and the reviewers. Any product that may be evaluated in this article, or claim that may be made by its manufacturer, is not guaranteed or endorsed by the publisher.
