# Supplementary material for: The Gut Microbiome of 54 Mammalian Species
Source: Front Microbiol. 2022 Jun 16;13:886252. doi: 10.3389/fmicb.2022.886252 (PMC9246093; doi:10.3389/fmicb.2022.886252)
Supplement: Supplementary file 1 [file Data_Sheet_1.zip › Data Sheet 1/Figure S1.docx]

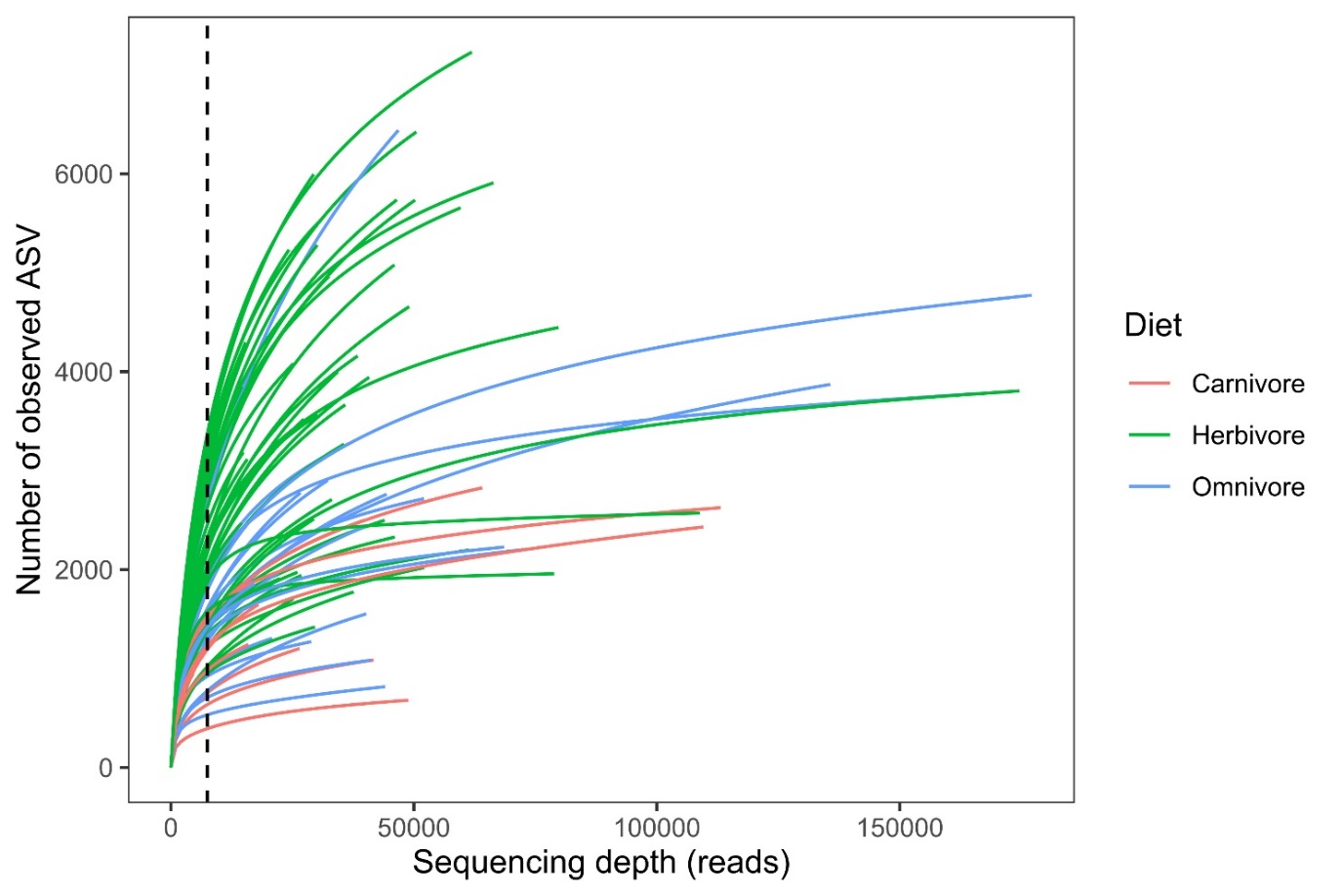


**Figure S1:** Rarefaction curve of all analysed animal microbiomes, coloured by diet. A broken line is drawn at 7,500 sequences, the minimum threshold for a sample to be included in the analysis. The individual samples are coloured by diet.
