# Supplementary material for: The Gut Microbiome of 54 Mammalian Species
Source: Front Microbiol. 2022 Jun 16;13:886252. doi: 10.3389/fmicb.2022.886252 (PMC9246093; doi:10.3389/fmicb.2022.886252)
Supplement: Supplementary file 1 [file Data_Sheet_1.zip › Data Sheet 1/Figure S2.docx]

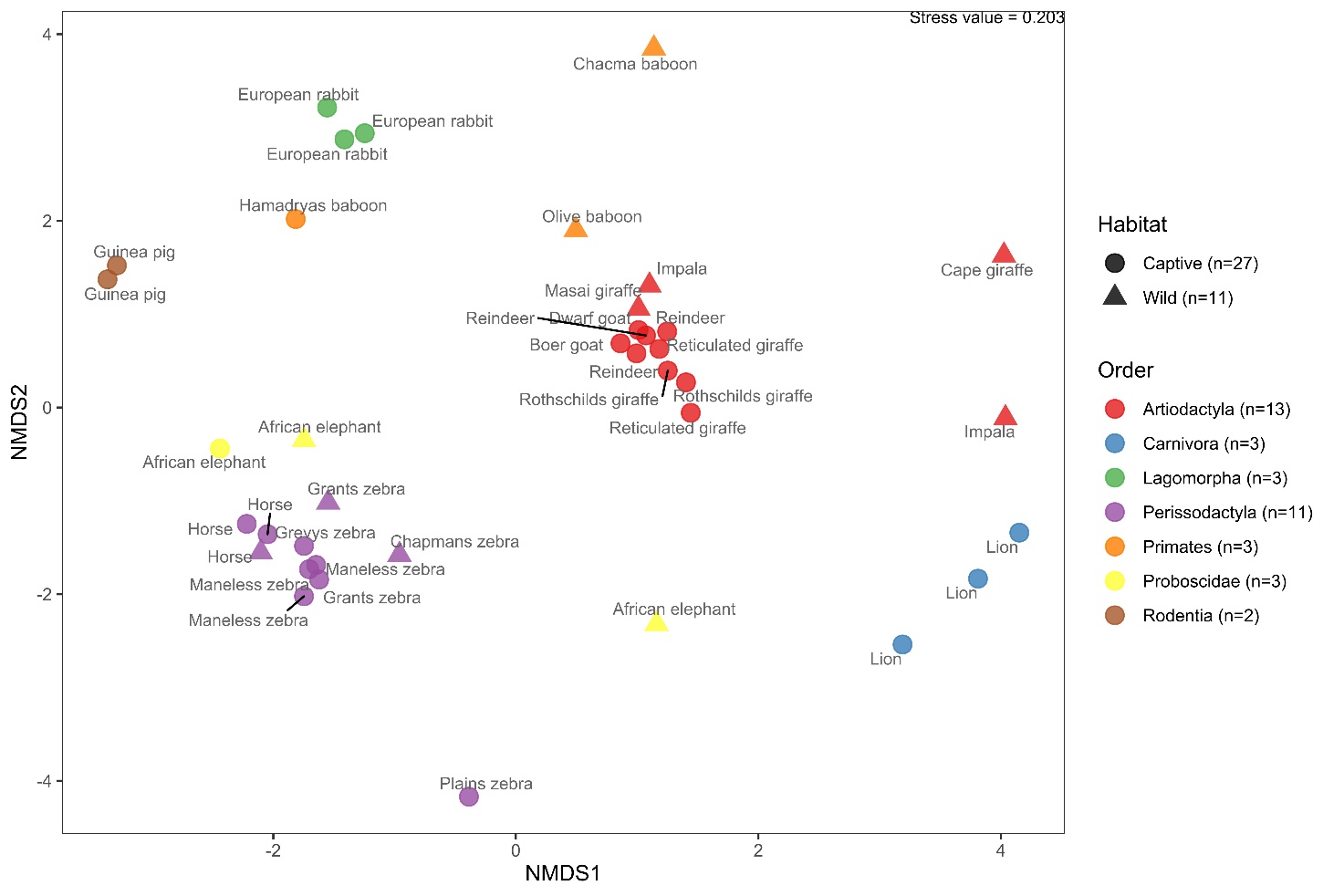


**Figure S2:** NMDS analysis on Bray-Curtis distances of all animals where more than one individual was sampled, regardless of captivity status. Samples are coloured by phylogeny, and shaped by captivity status.
