# Supplementary material for: The Gut Microbiome of 54 Mammalian Species
Source: Front Microbiol. 2022 Jun 16;13:886252. doi: 10.3389/fmicb.2022.886252 (PMC9246093; doi:10.3389/fmicb.2022.886252)
Supplement: Supplementary file 1 [file Data_Sheet_1.zip › Data Sheet 1/Figure S3.docx]

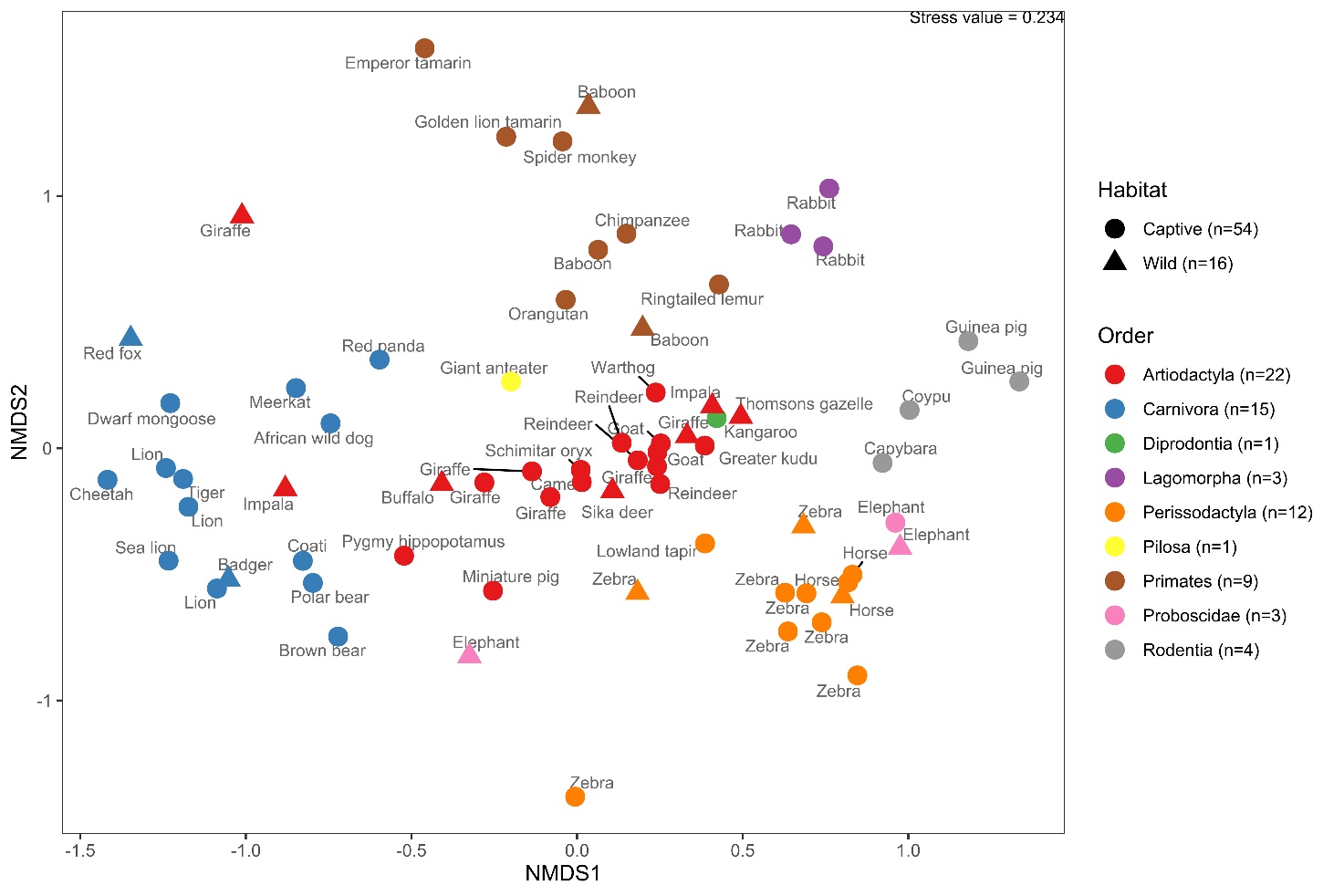


**Figure S3:** Gut microbiota in wild and captive animals. Beta diversity was measured using NMDS analysis on Bray-Curtis distances with samples coloured by phylogeny and shaped by captivity status. Simplified names of animals have been applied in the NMDS analysis to facilitate visibility.
