# Supplementary material for: The Gut Microbiome of 54 Mammalian Species
Source: Front Microbiol. 2022 Jun 16;13:886252. doi: 10.3389/fmicb.2022.886252 (PMC9246093; doi:10.3389/fmicb.2022.886252)
Supplement: Supplementary file 1 [file Data_Sheet_1.zip › Data Sheet 1/Figure S4.docx]

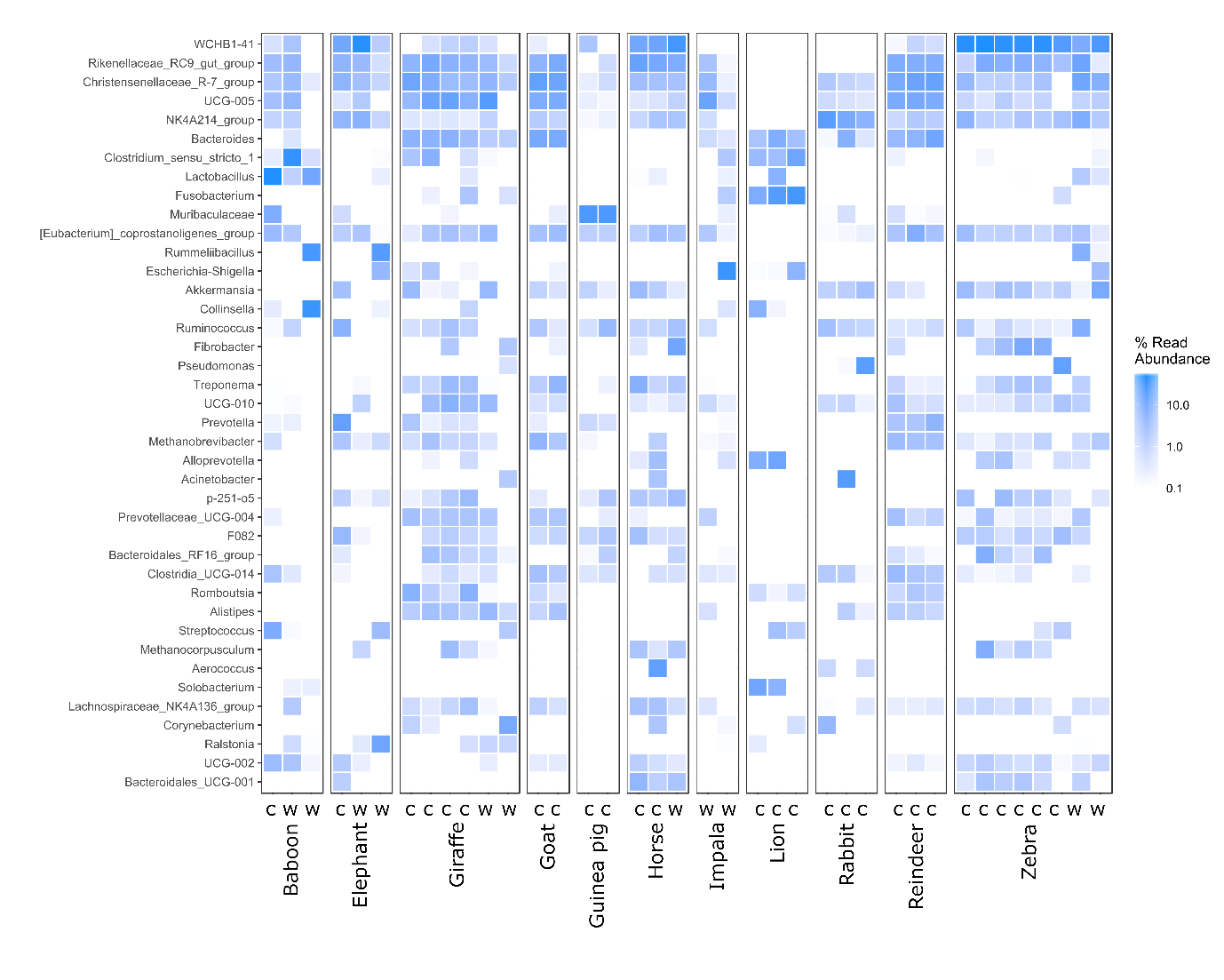


**Figure S4:** Heatmap of the 25 most abundant taxonomic groups at genus level, for all animal microbiomes where multiple samples for animal species could be analysed, regardless of captivity status. Animals are sorted by species, without taking subspecies into account. Captivity status is indicated with c for captive, and w for wild animals.
