# Supplementary material for: The Gut Microbiome of 54 Mammalian Species
Source: Front Microbiol. 2022 Jun 16;13:886252. doi: 10.3389/fmicb.2022.886252 (PMC9246093; doi:10.3389/fmicb.2022.886252)
Supplement: Supplementary file 1 [file Data_Sheet_1.zip › Data Sheet 1/Figure S5.docx]

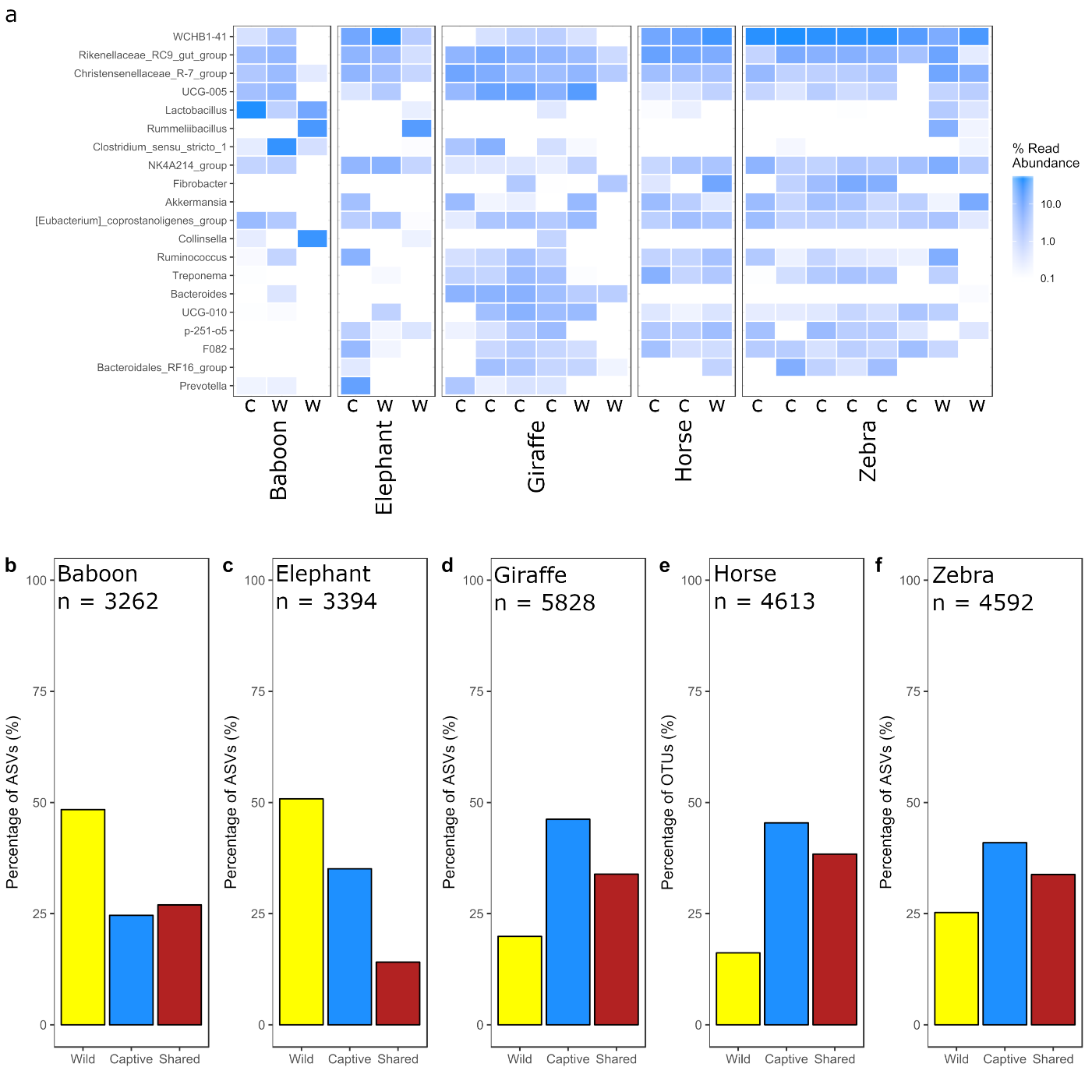


**Figure S5:** Comparison of microbiota profiles from wild and captive individuals of the same species, shown as heatmap (A) of the 20 most abundantly observed genera. The percentage (%) of ASVs observed either in the wild or captive specimens, or was observed in both (shared) for baboons (B), elephants (C), giraffes (D), horses (E) and zebras (F). In panel b-f the average estimated microbial community richness (Chao1) calculated per animal species is listed, and the captivity status is indicated with either c (captive) or w (wild).
