# Supplementary material for: The Gut Microbiome of 54 Mammalian Species
Source: Front Microbiol. 2022 Jun 16;13:886252. doi: 10.3389/fmicb.2022.886252 (PMC9246093; doi:10.3389/fmicb.2022.886252)
Supplement: Supplementary file 1 [file Data_Sheet_1.zip › Data Sheet 1/Table S1.docx]

**Table S1: Overview of all animals included in the study.**

| **Common name** | **Latin** | **Order** | **n captive** | | **n wild** | | **Diet** | **Gut morphology** |
| --- | --- | --- | --- | --- | --- | --- | --- | --- |
| African buffalo | *Syncerus caffer* | Artiodactyla | | 0 | | 1 | Herbivore | Foregut fermenting |
| African elephant | *Loxodonta africana* | Proboscidae | | 1 | | 2 | Herbivore | Hindgut fermenting |
| African wild dog | *Lycaon pictus* | Carnivora | | 1 | | 0 | Carnivore | Simple |
| Asiatic lion | *Panthera leo persica* | Carnivora | | 1 | | 0 | Carnivore | Simple |
| Bactrian camel | *Camelus bactrianus* | Artiodactyla | | 1 | | 0 | Herbivore | Foregut fermenting |
| Black-headed spider monkey | *Ateles fusciceps robustus* | Primates | | 1 | | 0 | Omnivore | Simple |
| Boer goat | *Capra hircus* | Artiodactyla | | 1 | | 0 | Herbivore | Foregut fermenting |
| Bornean orangutan | *Pongo pygmaeus* | Primates | | 1 | | 0 | Herbivore | Hindgut fermenting |
| Brown bear | *Ursus arctos* | Carnivora | | 1 | | 0 | Omnivore | Simple |
| California sea lion | *Zalophus californianus* | Carnivora | | 1 | | 0 | Carnivore | Simple |
| Cape giraffe | *Giraffa camelopardalis giraffa* | Artiodactyla | | 0 | | 1 | Herbivore | Foregut fermenting |
| Capybara | *Hydrochoerus hydrochaeris* | Rodentia | | 1 | | 0 | Herbivore | Hindgut fermenting |
| Chacma baboon | *Papio ursinus* | Primates | | 0 | | 1 | Omnivore | Simple |
| Chapmans zebra | *Equus quagga* | Perissodactyla | | 0 | | 1 | Herbivore | Hindgut fermenting |
| Cheetah | *Acinonyx jubatus* | Carnivora | | 1 | | 0 | Carnivore | Simple |
| Chimpanzee | *Pan troglodytes* | Primates | | 1 | | 0 | Omnivore | Simple |
| Common warthog | *Phacochoerus africanus* | Artiodactyla | | 1 | | 0 | Omnivore | Hindgut fermenting |
| Coypu | *Myocaster coypus* | Rodentia | | 1 | | 0 | Herbivore | Hindgut fermenting |
| Dwarf goat | *Capra hircus* | Artiodactyla | | 1 | | 0 | Herbivore | Foregut fermenting |
| Dwarf mongoose | *Helogale parvula* | Carnivora | | 1 | | 0 | Omnivore | Simple |
| Eastern grey kangaroo | *Macropus giganteus* | Diprotodontia | | 1 | | 0 | Herbivore | Foregut fermenting |
| Emperor tamarin | *Saguinus imperator* | Primates | | 1 | | 0 | Omnivore | Simple |
| European badger | *Meles meles* | Carnivora | | 0 | | 1 | Omnivore | Simple |
| European rabbit | *Oryctolagus cuniculus* | Lagomorpha | | 3 | | 0 | Herbivore | Hindgut fermenting |
| Giant anteater | *Myrmecophaga tridactyla* | Pilosa | | 1 | | 0 | Omnivore | Simple |
| Golden lion tamarin | *Leontopithecus rosalia* | Primates | | 1 | | 0 | Omnivore | Simple |
| Grants zebra | *Equus quagga boehmi* | Perissodactyla | | 1 | | 1 | Herbivore | Hindgut fermenting |
| Greater kudu | *Tragelaphus strepsiceros* | Artiodactyla | | 1 | | 0 | Herbivore | Foregut fermenting |
| Grevys zebra | *Equus grevyi* | Perissodactyla | | 1 | | 0 | Herbivore | Hindgut fermenting |
| Guinea pig | *Cavia porcellus* | Rodentia | | 2 | | 0 | Herbivore | Hindgut fermenting |
| Hamadryas baboon | *Papio hamadryas* | Primates | | 1 | | 0 | Omnivore | Simple |
| Horse | *Equus ferus caballus* | Perissodactyla | | 2 | | 1 | Herbivore | Hindgut fermenting |
| Impala | *Aepyceros melampus* | Artiodactyla | | 0 | | 2 | Herbivore | Foregut fermenting |
| Lion | *Panthera leo* | Carnivora | | 3 | | 0 | Carnivore | Simple |
| Lowland tapir | *Tapirus terrestris* | Perissodactyla | | 1 | | 0 | Herbivore | Hindgut fermenting |
| Maneless zebra | *Equus quagga borensis* | Perissodactyla | | 3 | | 0 | Herbivore | Hindgut fermenting |
| Masai giraffe | *Giraffa camelopardalis tippelskirchi* | Artiodactyla | | 0 | | 1 | Herbivore | Foregut fermenting |
| Meerkat | *Suricatta suricatta* | Carnivora | | 1 | | 0 | Omnivore | Simple |
| Miniature pig | *Sus scrofa domestica* | Artiodactyla | | 1 | | 0 | Omnivore | Simple |
| Olive baboon | *Papio anubis* | Primates | | 0 | | 1 | Omnivore | Simple |
| Plains zebra | *Equus quagga* | Perissodactyla | | 1 | | 0 | Herbivore | Hindgut fermenting |
| Polar bear | *Ursus maritimus* | Carnivora | | 1 | | 0 | Carnivore | Simple |
| Pygmy hippopotamus | *Hexaprotodon liberiensis* | Artiodactyla | | 1 | | 0 | Herbivore | Foregut fermenting |
| Red fox | *Vulpes Vulpes* | Carnivora | | 0 | | 1 | Omnivore | Simple |
| Red panda | *Ailurus fulgens* | Carnivora | | 1 | | 0 | Omnivore | Simple |
| Reindeer | *Rangifer tarandus* | Artiodactyla | | 3 | | 0 | Herbivore | Foregut fermenting |
| Reticulated giraffe | *Giraffa camelopardalis reticulata* | Artiodactyla | | 2 | | 0 | Herbivore | Foregut fermenting |
| Ringtailed lemur | *Lemur catta* | Primates | | 1 | | 0 | Omnivore | Simple |
| Rothschilds giraffe | *Giraffa camelopardalis rothschildii* | Artiodactyla | | 2 | | 0 | Herbivore | Foregut fermenting |
| Scimitar oryx | *Oryx dammah* | Artiodactyla | | 1 | | 0 | Herbivore | Foregut fermenting |
| Siberian tiger | *Panthera tigris altaica* | Carnivora | | 1 | | 0 | Carnivore | Simple |
| Sika deer | *Cervus nippon* | Artiodactyla | | 0 | | 1 | Herbivore | Foregut fermenting |
| South american coati | *Nasua nasua* | Carnivora | | 1 | | 0 | Omnivore | Simple |
| Thomsons gazelle | *Eudorcas thomsonii* | Artiodactyla | | 0 | | 1 | Herbivore | Foregut fermenting |
