# Supplementary material for: The Gut Microbiome of 54 Mammalian Species
Source: Front Microbiol. 2022 Jun 16;13:886252. doi: 10.3389/fmicb.2022.886252 (PMC9246093; doi:10.3389/fmicb.2022.886252)
Supplement: Supplementary file 1 [file Data_Sheet_1.zip › Data Sheet 1/Table S2.docx]

**Table S2: Alpha diversity values for all captive animals.**

| **Animal** | **Reads** | **Observed ASVs** | **Chao1** | **Ratio Obs/Chao1** |
| --- | --- | --- | --- | --- |
| Horse | 9420 | 2441 | 4089 | 0.60 |
| Reindeer | 9658 | 3609 | 5644 | 0.64 |
| Maneless zebra | 14534 | 2470 | 3255 | 0.76 |
| Reticulated giraffe | 14613 | 3852 | 5422 | 0.71 |
| Maneless zebra | 14894 | 3186 | 4344 | 0.73 |
| Rothschilds giraffe | 15357 | 4297 | 6113 | 0.70 |
| Lion | 15965 | 1244 | 1668 | 0.75 |
| Siberian tiger | 17993 | 1647 | 2194 | 0.75 |
| Emperor tamarin | 20837 | 1305 | 1570 | 0.83 |
| Golden lion tamarin | 23061 | 1800 | 2355 | 0.76 |
| Reindeer | 24297 | 5233 | 6894 | 0.76 |
| Coypu | 25182 | 1706 | 2830 | 0.60 |
| Maneless zebra | 25197 | 4081 | 5349 | 0.76 |
| European rabbit | 26044 | 1974 | 2680 | 0.74 |
| Polar bear | 26479 | 1203 | 1764 | 0.68 |
| Pygmy hippopotamus | 26864 | 1942 | 2517 | 0.77 |
| Grevys zebra | 28828 | 3543 | 4816 | 0.74 |
| South american coati | 28915 | 1272 | 1602 | 0.79 |
| Schimitar oryx | 29398 | 5995 | 7957 | 0.75 |
| Guinea pig | 29657 | 1420 | 2017 | 0.70 |
| Grants zebra | 30260 | 3565 | 4748 | 0.75 |
| Dwarf goat | 31059 | 5545 | 6871 | 0.81 |
| Common warthog | 32266 | 2902 | 4105 | 0.71 |
| Greater kudu | 32558 | 4966 | 6641 | 0.75 |
| African elephant | 33131 | 2709 | 3941 | 0.69 |
| Bornean orangutan | 35656 | 3268 | 4379 | 0.75 |
| Lowland tapir | 35871 | 3666 | 4979 | 0.74 |
| Meerkat | 36678 | 3275 | 4179 | 0.78 |
| Horse | 38432 | 4159 | 5562 | 0.75 |
| African wild dog | 39680 | 2498 | 3250 | 0.77 |
| Hamadryas baboon | 40232 | 2441 | 3424 | 0.71 |
| Capybara | 43988 | 2499 | 3338 | 0.75 |
| Brown bear | 44088 | 817 | 1242 | 0.66 |
| Ringtailed lemur | 44407 | 2762 | 3783 | 0.73 |
| European rabbit | 46074 | 2329 | 2973 | 0.78 |
| Bactrian camel | 46499 | 5737 | 7571 | 0.76 |
| Giant anteater | 46806 | 6440 | 9816 | 0.66 |
| California sea lion | 48835 | 680 | 862 | 0.79 |
| Eastern grey kangaroo | 49046 | 4658 | 6552 | 0.71 |
| Rothschilds giraffe | 50252 | 5735 | 7476 | 0.77 |
| Reindeer | 50500 | 6423 | 7954 | 0.81 |
| Red panda | 52078 | 2717 | 3177 | 0.86 |
| Guinea pig | 52121 | 2021 | 2823 | 0.72 |
| European rabbit | 61358 | 2198 | 2726 | 0.81 |
| Boer goat | 61939 | 7230 | 8649 | 0.84 |
| Lion | 64079 | 2827 | 3466 | 0.82 |
| Reticulated giraffe | 66382 | 5909 | 6792 | 0.87 |
| Dwarf mongoose | 72584 | 2203 | 2658 | 0.83 |
| Plains zebra | 108931 | 2572 | 2699 | 0.95 |
| Lion | 109558 | 2431 | 3419 | 0.71 |
| Cheetah | 113176 | 2626 | 3149 | 0.83 |
| Miniature pig | 135711 | 3872 | 5141 | 0.75 |
| Black-headed spider monkey | 166312 | 3779 | 4257 | 0.89 |
| Chimpanzee | 177147 | 4773 | 5577 | 0.86 |
